# Induction of G2M Arrest by Flavokawain A, a Kava Chalcone, Increases the Responsiveness of HER2-Overexpressing Breast Cancer Cells to Herceptin

**DOI:** 10.3390/molecules22030462

**Published:** 2017-03-14

**Authors:** Danielle D. Jandial, Lauren S. Krill, Lixia Chen, Chunli Wu, Yu Ke, Jun Xie, Bang H. Hoang, Xiaolin Zi

**Affiliations:** 1 Department of Obstetrics & Gynecology, University of California, Irvine, Orange, CA 92868, USA; djandial@uci.edu (D.D.J.); lsmithkr@uci.edu (L.S.K.); 2 Department of Urology, University of California, Irvine, Orange, CA 92868, USA; syzyclx@163.com (L.C.); kedi2009@126.com (C.W.); ky@zzu.edu.cn (Y.K.); xiej@uci.edu (J.X.); 3 Department of Orthopedic Surgery, Montefiore Medical Center, Albert Einstein College of Medicine, Bronx, NY 10476, USA; bahoang@montefiore.org; 4 Department of Pharmacology, University of California, Irvine, Orange, CA 92868, USA

**Keywords:** Flavokawain A, HER2 overexpression, resistance to apoptosis

## Abstract

HER2/neu positive breast tumors predict a high mortality and comprise 25%–30% of breast cancer. We have shown that Flavokawain A (FKA) preferentially reduces the viabilities of HER2-overexpressing breast cancer cell lines (i.e., SKBR3 and MCF7/HER2) versus those with less HER2 expression (i.e., MCF7 and MDA-MB-468). FKA at cytotoxic concentrations to breast cancer cell lines also has a minimal effect on the growth of non-malignant breast epithelial MCF10A cells. FKA induces G2M arrest in cell cycle progression of HER2-overexpressing breast cancer cell lines through inhibition of Cdc2 and Cdc25C phosphorylation and downregulation of expression of Myt1 and Wee1 leading to increased Cdc2 kinase activities. In addition, FKA induces apoptosis in SKBR3 cells by increasing the protein expression of Bim and BAX and decreasing expression of Bcl_2_, Bcl_X/L_, XIAP, and survivin. FKA also downregulates the protein expression of HER-2 and inhibits AKT phosphorylation. Herceptin plus FKA treatment leads to an enhanced growth inhibitory effect on HER-2 overexpressing breast cancer cell lines through downregulation of Myt1, Wee1, Skp2, survivin, and XIAP. Our results suggest FKA as a promising and novel apoptosis inducer and G2 blocking agent that, in combination with Herceptin, enhances for the treatment of HER2-overexpressing breast cancer.

## 1. Introduction

Conventional chemo-/radio-therapies for breast cancer non-specifically cause deleterious effects to healthy tissues and have been associated with significant side effects, including cardiotoxicity [1,2]. In this regard, some phytochemicals are either part of the human diet or consumed as dietary supplements and do not show adverse health effects even at large doses [3]. Phytochemicals that interact with tumor-specific targets to promote anticancer effects, but remain non-toxic at therapeutic levels may, therefore, represent attractive new dimensions in management of breast cancer.

Human epidermal-growth-factor receptor 2 (HER2) and estrogen receptor (ER) are two breast tumor-specific targets [4,5]. HER2 breast tumors predict a high mortality and comprise 25%–30% of breast cancer [6]. The use of trastuzumab (Herceptin, a blocking antibody for HER2 signaling) has demonstrated clinical benefits in the management of HER2 positive metastatic breast cancer [7]. However, the outcome of this therapy in metastatic breast cancer remains unsatisfying due to frequent development of resistance to trastuzumab and risks of cardiac dysfunctions [7,8]. HER2 could also be a target for breast cancer prevention because HER2 is overexpressed in a majority of ductal carcinoma in situ (DCIS) cases of the breast, a non-obligate precursor of invasive breast cancer [9,10,11]. At least 50% of HER2-overexpressing breast cancer is ER-positive at baseline [12]. Tamoxifen is the most commonly used drug for the treatment of ER-positive breast cancer and has been shown to be effective in prevention [5,13]. However, HER2-overexpressing ER-positive tumors can develop resistance to tamoxifen by the acquisition of tamoxifen-stimulated growth [14]. Thus, new therapeutic or preventive approaches for HER2-overexpressing breast cancers are urgently needed.

Flavokawain A (FKA) is the predominant kava chalcone that constitutes up to 0.46% of the kava extract. FKA was first by our group to be a potent apoptosis inducer in screening studies for growth inhibition of many types of cancer cell lines, including bladder, breast, colon, liver, and prostate cancers, melanoma, sarcoma, etc. [15,16,17,18,19,20]. FKA at concentrations which significantly inhibit the growth of cancer cell lines has minimal effect on the growth of normal cells [18]. FKA activates both death-receptor and the mitochondria-mediated apoptotic pathways through increasing the expression of pro-apoptotic proteins DR5, Bim, and Bax, and decreasing the expression of anti-apoptotic proteins Bcl2, Bcl_X/L_, surviving, and XIAP [15,16]. We have further demonstrated that FKA is a novel neddylation inhibitor and causes degradation of Skp2 protein [18,20]. Dietary supplementation with FKA inhibits tumorigenesis in transgenic mouse models of bladder and prostate cancer, without exhibiting any adverse effects on major organ function (including liver function) and homoeostasis of the mice [18,20,21,22]. However, the effect of FKA on HER2-overexpressing breast cancer has not yet been reported. In this study, we have shown that FKA preferentially inhibits the growth of HER2-overexpressing breast cancer cell lines versus those with less HER2 expression by downregulating the expression of Myt1 and Wee1 kinases leading to reduced phosphorylation levels of Cdc2, which then leads to G2M arrest and apoptosis. In addition, FKA enhances the growth inhibitory effect of Herceptin on Her2-overexpressing breast cancer cells.

## 2. Results

FKA preferentially inhibits the anchorage-dependent and independent growth of HER2-overexpressing breast cancer cell lines.

**Figure 1:**
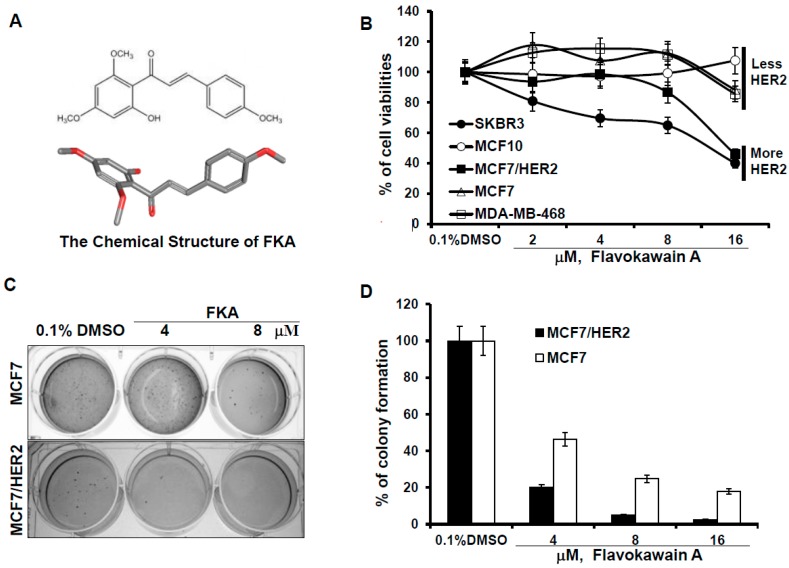
FKA inhibits the anchorage-dependent and independent growth of breast cancer cell lines with minimal effect on normal breast epithelial cells. (**A**) the 2D and 3D chemical structure of FKA; (**B**) 5 × 10^4^ MCF10A, MCF7, MCF7/HER2, MDA-MB-468, and SKBR3 cells were plated in 24-well culture plates. After 72 h, cells were treated with 0.05% dimethyl sulfoxide DMSO or FKA at the indicated concentrations. After 72 h of treatment, cell densities were measured by MTT assay. Points show the mean of four independent plates; bars, SE. Each sample was counted in duplicate; (**C**) MCF7 and MCF7/HER2 cells were grown in soft agar in six-well plates and treated with 0.05% DMSO or FKA at the indicated concentrations for 30 days. The number of colonies was determined by counting them under an inverted phase-contrast microscope at ×100 magnification; a group of >10 cells was counted as a colony; and (**D**) quantitative analysis of inhibitory effect of FKA against soft agar colony formation. Columns show the means of four independent wells at 30 days after the start of cell seeding; bars, SE.

To determine whether FKA specifically inhibits the growth of breast cancer cells, including HER2-overexpressing breast cancer cells, an immortalized normal breast epithelial cell line, MCF10A, ER-positive (MCF7) and ER-negative (MDA-MB-468) breast cancer cell lines with less HER2 expression, and ER-positive (MCF7/HER2) and ER-negative (SKBR3) breast cancer cell lines with HER2-overexpression were examined. SKBR3 was isolated from the pleural effusion of a 43-year-old female with metastatic ductal adenocarcinoma of the breast with HER2/neu amplification [23]. MCF10A was originally derived from benign breast tissue from a woman with fibrocystic disease [24]. MCF/HER2 is a MCF7 clone engineered to overexpress HER2 [25]. Figure 1B shows that FKA inhibited the growth of MCF7, MDA-MB-468, SKBR3, and MCF7/HER2 cells in a dose-dependent manner. At the same concentrations, FKA did not cause any noticeable inhibition on the growth of the MCF10A cell line (Figure 1B). Moreover, FKA treatment for 24 h more effectively inhibited the growth of SKBR3 and MCF-7/HER2 cell lines which express more HER2 than either MCF7 or MCF10A which express less HER2 (Figure 1B). The IC_50_ values of FKA on the growth of HER2 overexpressing SKBR3 and MCF-7/HER2 cells are 10 and 13.6 μM, respectively, versus 38.4 and 45 μM for MCF7 and MDA-MB-468 cells, respectively (Table 1) (*Ps* < 0.05).

Anchorage-independent growth in suspension media, such as soft agar, is an important measure of transformation (Figure 1). FKA treatment resulted in a greater decrease in the colony formation of MCF/HER2 than that of its parental cell line MCF7 (Figure 1C,D). It appears that FKA is more effective in the inhibition of colony formation than cell growth in attached cell culture conditions. FKA at a concentration of 4 μM inhibits the colony formation of MCF/HER2 and MCF7 by 80% and 54%, respectively (Figure 1D). Together, these results suggested that FKA can specifically inhibit HER2-overexpressing breast cancer cells with minimal effect on normal breast epithelial cells.

### 2.1. The Effect of FKA on Cell Cycle Progression Differs between HER2 Overexpressing versus Low-Expressing Breast Cancer Cell Lines

**Figure 2:**
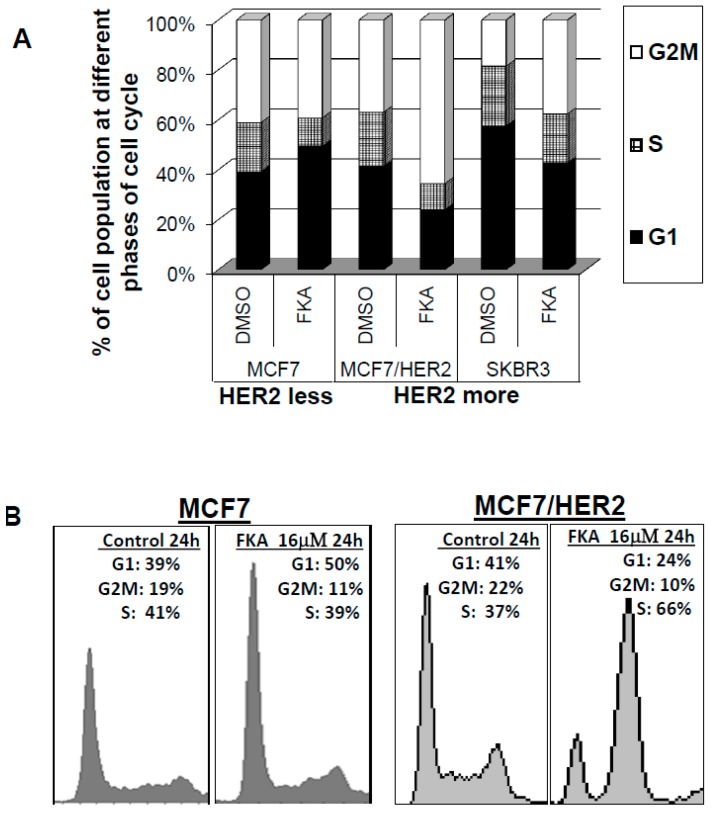
FKA induces G2M arrest in HER2-overexpressing MCF7/HER2 and SKBR3 cells and G1 arrest in HER2 less MCF7 cells. MCF7, MCF7/HER2, and SKBR3 cells were treated with 0.05% DMSO or 16 μM FKA for 24 h. Cell cycle population was determined by FACS analysis. (**A**) Quantitative analysis of the percentage of cell cycle phase; and (**B**) graphical presentation of cell cycle distribution after FKA treatment.

To examine whether the cell growth inhibitory effects of FKA were induced via perturbation in cell cycle progression, we performed fluorescence-activated cell sorting analysis of control (0.1% DMSO) and 16 μM FKA–treated cells. Figure 2A,B indicated a G1 arrest in p53 wild-type and HER2 less MCF7 cells treated with FKA (G1 population, 39.2% for control versus 49.5% for FKA at 24 h of treatments; Student’s *t*-test, *Ps* < 0.01), For HER2-overexpressing, but p53 wild-type MCF7/HER2 cells, as well as HER2-overexpressing and p53 mutant SKBR3 cells, FKA at the same concentration induced a significant G2-M arrest (G2-M population, 36.9% and 18.5% for control versus 65.5% and 37.7% for FKA treatments of MCF7/HER2 and SKBR3 cells, respectively, for 24 h; Student’s *t*-test, *Ps* < 0.01) (Figure 2A,B). These results indicate that the growth-inhibitory effects of FKA on HER2-overexpressing or minimally-expressing breast cancer cells is associated with a G1 or M phase arrest, respectively, and that the FKA induced G2M arrest in HER2-overexpressing breast cancer cells is independent of p53 status.

### 2.2. The Mechanisms of FK A–Induced G2M Arrest in HER2-Overexpressing SKBR3 Cells Are Associated with Inhibition of Cdc2 Phosphorylation via Downregulation of Wyt1 and Wee1 Expression and Cdc25C Phosphorylation

**Figure 3:**
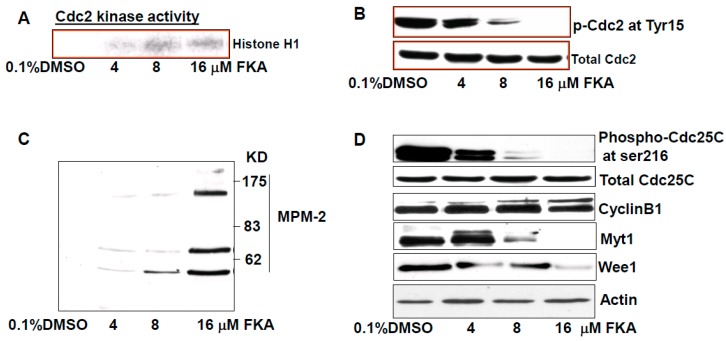
FKA increases MPM-2 phosphorylation and Cdc kinase activity via inhibition of Cdc2 and Cdc25 phosphorylation and downregulation of Myt1 and Wee1 expression. (**A**) Cdc2 associated histone H1 kinase activity was decreased dose-dependently by FKA treatment for 24 h in SKBR3 cells; (**B**–**D**) SKBR3 cells were treated with indicated concentrations of FKA for 24 h. Phosphorylation levels of Cdc2, MPM-2, and Cdc25C as well as protein levels of cyclin B1, Myt1, and Wee1 were detected by Western blotting analysis. β-actin was detected as a loading control. A representative blot was shown from three independent experiments.

Figure 3A shows that FKA treatment resulted in a dose-dependent increase in Cdc2 kinase activity. Cdc2 kinase that is considered a driving force of G2M transition and is activated by dephosphorylation of Cdc2 at Tyr15 [26]. HER2 was shown to bind to Cdc2 and phosphorylate Cdc2 at Tyr15, leading to a delay in G2M transition [26]. Figure 3B shows that FKA treatment decreased the phosphorylation levels of Cdc2 at the Tyr15 site in a dose-dependent manner without a change in Cdc2 protein expression. We examined the reactivity of the MPM-2 antibody—an antibody specific for its preferential reactivity towards mitotic versus interphase cells, and can react with subsets of proteins that are phosphorylated upon entry into mitosis [27], we observed that FKA increased the expression of mitotic phosphoproteins (Figure 3C), which confirmed an M phase arrest by FKA. The decrease in Cdc2 phosphorylation at Tyr15 after FKA treatments was accompanied by reduced expression of Cdc2 inhibitors Wee1 and Myt1 and dephosphorylation of Cdc25C. FKA treatment did not affect the expression of Cyclin B1. Taken together, these results suggest that FKA activated Cdc25C via its dephosphorylation at Ser216 and decreased the expression of Cdc2 inhibitors that promote mitosis via dephosphorylation of Cdc2 at Tyr15 leading to enhancement of Cdc2 kinase activity.

### 2.3. FKA Induces Apoptosis in HER2-Overexpressing Breast Cancer SKBR3 Cells

**Figure 4:**
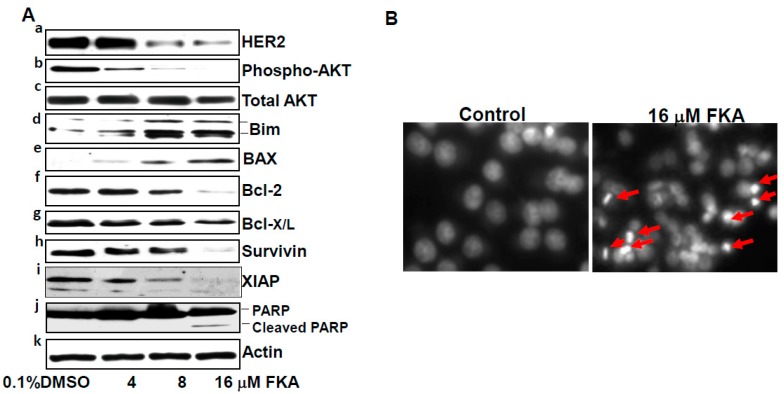
FKA induces apoptosis in HER2-overexpressing SKBR3 breast cancer cell lines via downregulation of HER2, phosphorylated AKT, Bcl2, Bcl-_X/L_, survivin, and XIAP and upregulation of Bim and BAX. (**A**) SKBR3 cells were treated with indicated concentrations of FKA for 24 h. Western blotting analysis of HER2, phosphorylated AKT, AKT, Bim, BAX, Bcl-2, Bcl-_X/L_, survivin, XIAP, and PARP is shown by a representative blot from three experiments. β-actin was detected as a loading control; and (**B**) DAPI staining of nuclear morphology under fluorescence microscope (magnification: 200×). Arrows indicate cells with nuclear condensation and fragmentation, which were counted as apoptotic cells.

We further examined the mechanisms by which FKA is more effective in inhibiting the growth of HER2-overexpressing breast cancer cell lines. We first examined whether FKA can regulate the receptor levels of HER2 in HER2-overexpressing SKBR3 cells. Figure 4A panel a shows that Flavokawain A caused a dose-dependent decrease in the protein levels of HER2. HER2 overexpression was reported to cause resistance to apoptosis by increasing the expression of antiapoptotic proteins (e.g., survivin, Bcl-2, and Bcl-_X/L_) and decreasing the expression of a proapoptotic protein Bim via activation of the AKT mediated survival pathway [26,28,29,30,31]. Therefore, we examined whether FKA can affect the expression of these HER2-modulated molecules in SKBR3 cells. FKA treatment resulted in decreased levels of AKT phosphorylation without change in its protein levels (Figure 3A, panels b and c). In addition, FKA increased the expression of pro-apoptotic proteins Bim and Bax and decreased the expression of anti-apoptotic proteins survivin, XIAP, Bcl-2, and Bcl-_X/L_ in a dose-dependent manner (Figure 3A, panels d–i). As a result of these alterations, FKA induced a cleavage of PARP protein, as well as nuclear fragmentation and condensations, hallmarks for apoptosis (Figure 4Aj,B). These results suggested that FKA exhibit a robust mechanism in induction of apoptosis in HER2-overexpressing SKBR3 cells.

### 2.4. Herceptin and FKA Combination Causes Enhanced Growth Inhibitory Effect on HER2-Overexpressing Breast Cancer Cells via Down-Regulation of Myt1, Wee1, Survivin, and XIAP Expression

**Figure 5:**
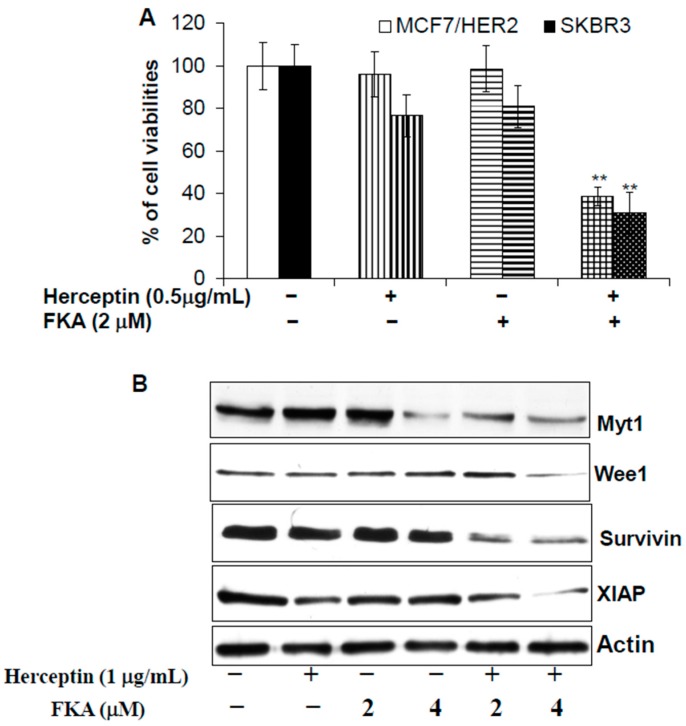
FKA plus Herceptin results in enhanced growth inhibitory effect on HER2-overexpressing breast cancer cell lines via downregulation of Myt1, Wee1, surviving, and XIAP. (**A**) MCF7/HER2 and SKBR3 cells (5 × 10^4^) were plated in 24-well culture plates. After 24 h, cells were treated with 0.05% DMSO, Herceptin alone, FKA alone, and FKA plus Herceptin, at the indicated concentrations. After 72 h of treatment, cell densities were measured by MTT assay. Points represent the mean of four independent plates; bars, SE. Each sample was counted in duplicate. “**” denotes “*p <* 0.01” for Hercepin or FKA alone vs the combination; (**B**) SKBR3 cells were treated with indicated concentrations of Herceptin, FKA, or their combination for 24 h. Western blotting analysis of Myt1, Wee1, survivin, and XIAP is shown by a representative blot from three experiments. β-actin was detected as a loading control.

Herceptin is a major drug for treatment of HER2 positive and metastatic breast cancer and can improve patients’ overall survival [32]. However, development of resistance to Herceptin is common and the treatment is associated with cardiac dysfunction in 2%–7% of cases [32]. Therefore, there is a need for a novel agent that can improve both the efficacy and safety of Herceptin treatment through combination with a complementary agent. As shown in Figure 5A, Herceptin, in combination with FKA, reduced the viabilities of MCF7/HER2 and SKBR3 cells by 61% and 69%, respectively, whereas 2 μM FKA alone only decreased the viabilities by 1% and 19%, respectively, and 0.5 mg/mL Herceptin, by 4% and 23%, respectively (Figure 5A). These results suggest that FKA and Herceptin may act synergistically to inhibit the growth of HER2-overexpressing breast cancer cells. Further experiments show that FKA enhanced the inhibitory effects of Herceptin on the protein expression of Myt1, Wee1, Survivin and XIAP (Figure 5B). The combined effect of Herceptin and FKA on downregulation of Cdc2 inhibitors (i.e., Myt1 and Wee1) and inhibitors of apoptosis (i.e., survivin and XIAP) is likely attributable to the enhanced growth inhibition of HER2-overexpressing breast cancer cells by these two agents.

## 3. Discussion

The genetic and molecular heterogeneity of breast cancer represents a challenge for treatment and prevention strategies and, thus, the development of innovative targeted therapies for specific tumor subtypes is crucial [33]. Overexpression of HER2 defines a subtype of breast cancer that is associated with a poor clinical outcome [34], which is a critical therapeutic target. Phase III clinical trials [35,36] have demonstrated the efficacy of the humanized HER2 antibody Herceptin as a single agent for treatment of advanced breast cancer, while also improving survival when used as a first-line therapy in combination with chemotherapy. In addition, Herceptin has the potential for improving the outcomes among women with HER2-positive early breast cancer [37]. Nevertheless, the outcome of current therapies for HER2-positive breast cancer remains unsatisfying, as only a fraction of patients respond successfully to Herceptin therapy and the risk of recurrence remains high. In addition, an increased risk of cardiac dysfunction associated with Herceptin therapy requires stringent criteria for selection of patients [7]. In this study, we have demonstrated that FKA, a kava chalcone, preferentially inhibited the growth of HER2-overexpressing breast cancer cells with a minimal effect on the growth of non-malignant breast epithelial cells. The growth inhibitory effect of FKA on HER2-overexpressing breast cancer cells is associated with a G2M arrest in cell cycle progression and is associated with induction of apoptosis. In addition, Herceptin plus FKA treatment led to enhanced inhibitory effects on the growth of HER2-overexpressing breast cancer cells. The growth inhibition by treatment with Herceptin plus FKA is greater than the sum of either agent alone. These results indicates that FKA with an excellent safety profile [18,21] deserves further investigation for its potential use as an adjuvant agent in combination with Herceptin for treatment of HER2-positive breast cancer or as a preventative agent for targeting HER2-positive early breast cancer in preventing its recurrence and progression.

Overexpression of HER2 commonly causes the resistance of apoptosis by increasing anti-apoptotic proteins (e.g., Bcl-2, Bcl-_X/L_, Mcl-1, and survivin) and decreasing the proapoptotic protein Bim and, thus, contributes to tumor progression and contributes to the resistance to chemotherapy [9,28,29,30,31]. The underlying mechanism(s) responsible for the antiapoptotic effects of ErbB2 overexpression may be due to the concomitant up-regulation of the PI3K-Akt/NF-κB survival pathway [29,38]. We have shown here that FKA inhibits the PI3K/AKT pathway by downregulation of HER2 protein expression and by dephosporylation of AKT. In addition, Kwon et al. [39] reported that FKA blocked the lipopolysaccharides-induced activation of NF-κB in RAW 264.7 macrophages. Consistent with our previously-published results in prostate and bladder cancer cell lines [15,16,17,18], we also have shown that FKA exhibited a robust mechanism of inducing apoptosis in HER2-overexpressing breast cancer cell lines by increasing the expression of proapoptotic proteins Bim and BAX and decreasing anti-apoptotic proteins (i.e., Bcl-2, Bcl-_X/L_, XIAP, and survivin). Therefore, the accumulating evidence supports that FKA can counteract the HER2 overexpression mediated apoptosis resistance by inhibiting the PI3K-Akt/NF-κB survival pathway, which leads to upregulation of proapoptotic proteins and downregulation of anti-apoptotic proteins.

Cell cycle progression through G1 phase, S phase (DNA replication), G2 phase, and M phase (mitosis and cytokinesis) is essential for cell growth. However, there are also two important cell cycle checkpoints at G1 and G2 phases, respectively, for controlling unlimited growth [40]. Cancer cells commonly develop defective G1 checkpoint via loss of tumor suppressors (e.g., p53, and RB), where the G2 checkpoint is often intact and left to be critical in cancer cell survival [40]. HER2 overexpression or activation has been shown to activate the G2 checkpoint in breast cancer cells by directly interacting with Cdc2 phosphatase and then phosphorylating Cdc2 at Tyr15 and by decreasing Cdc2 kinase activity [26,41]. Therefore, HER2-overexpressing breast cancer often has a higher level of Cdc2 phosphorylation at Tyr15, which may be particularly susceptible to agents that can abrogate the G2 checkpoint. We show here that FKA inhibits Cdc2 phosphorylation, increases Cdc2 kinase activity, and induces G2M arrest in HER2-overexpressing breast cancer cell lines by both (1) de-phosphorylation of Cdc25C and (2) downregulation of Cdc2 inhibitors: Myt1 and Wee1. Similarly, we previously reported that FKA selectively induces G2M arrest and growth inhibitory effect on p53 and pRb defective bladder and prostate cancer cell lines [16,18]. In addition, FKA enhanced the growth inhibitory effect of HER2 specific antibody Herceptin on HER2-overexpressing breast cancer cell line by down-regulation of Myt1, Wee1, survivin, and XIAP.

S-phase kinase-associated protein 2 (Skp2) is the F-box component of an E3 ubiquitin ligase complex, which recognizes and degrades many substrates, such as p27, MacroH2A1, etc., for promoting cell cycle progression [42]. Skp2 depletion in melanoma cells and mouse embryonic fibroblasts (MEFs) results in G2M arrest and accumulation of polyploid cell forms [43,44]. More importantly, Skp2 deletion in a Skp2 knockout mouse model has been shown by multiple groups to markedly restrict tumorigenesis under different conditions of tumor initiation and promotion, including PTEN, ARF, pRB inactivation, as well as HER-2/Neu overexpression [45,46]. Furthermore, Skp2 deletion was associated with Herceptin sensitivity and suppression of Skp2 expression sensitized HER2-positive breast tumors to Herceptin treatment [47,48]. Our recent publication also has demonstrated that FKA acts as a neddylation inhibitor leading to degradation of Skp2 and inhibition of prostate tumorigenesis in the TRAMP model [18]. In our studies, FKA downregulated the protein expression of Skp2 in all tested cancer cell lines that were derived from prostate, breast, renal, liver, lung, colon, and cervical cancers, melanoma and osteosarcoma, regardless of their genetic background [18]. Therefore, the downregulation of Skp2 expression may also contribute to the FKA induced G2M arrest and cell growth inhibition in HER2-overexpressing breast cancer. Further in vivo testing of FKA’s effect on HER2 driven mammary carcinogenesis in a transgenic model and tumor growth in a xenograph model is warranted.

**Figure 6:**
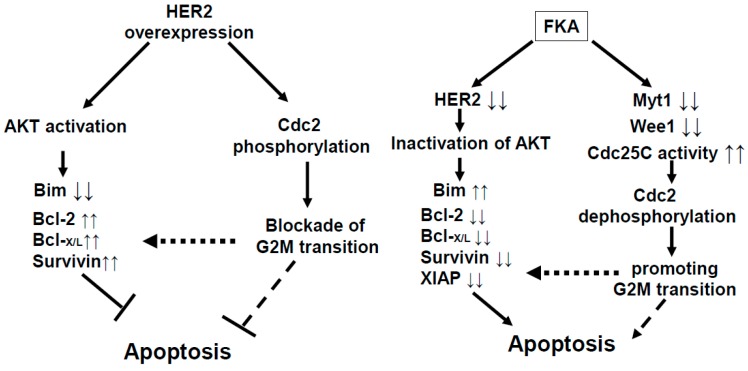
Model of a mechanism by which FKA counteracts HER2-mediated apoptosis resistance and functions as a selective G2 abrogator in HER2-overexpressing breast cancer cells.

Our results in this study can be summarized as in Figure 6. HER-2 overexpression activates AKT, suppresses the expression of apoptotic protein Bim, and increases the expression of antiapoptotic proteins Bcl2, Bcl-_X/L_, and survivin, which then causes resistance to apoptosis. In addition, Her2 overexpression directly phosphorylates Cdc2 and inactivates Cdc2, which delays G2M transition and then increases the survival of cancer cells. We have shown that FKA can counteract the effect of HER2 overexpression-mediated resistance to apoptosis by inactivating AKT, with a subsequent increase of Bim expression and decrease of Bcl2, Bcl-_X/L_, XIAP, and survivin; and by activating Cdc25C and downregulating Myt1 and Weel, which dephosphorylates and activates Cdc2 to promote G2M transition and premature mitosis. Therefore, FKA suggests its usefulness as a “selective G2M abrogator” aiming to target HER2-overexpressing breast cancer and may be a sensitizer for Herceptin-based therapies.

## 4. Materials and Methods

### 4.1. Cell Lines, Compounds, and Reagents

The SKBR3, MCF10A, MCF7, and MDA-MB-468 cell lines were obtained from American Type Culture Collection (ATCC, Manassas, VA, USA). All cell lines used in this study were within 20 passages after receipt. The cell lines were tested and authenticated by ATCC. MCF-7/HER2 cells [25] were provided by Dr. Alaoui-Jamali (McGill University). The MCF-10A cell line was grown in MEGM Bulletkit media. The MCF7, SKBR3, and MDA-MB-468 cell lines were cultured and passaged in minimum essential Eagle’s medium, McCoy’s 5A medium and Leibovitz’s L-15 medium, respectively, supplemented with 10% fetal bovine serum (FBS). MCF-7/HER2 cells were maintained in EMEM medium with 10% FBS and 400 μg/mL G418 at 37 °C and 5% CO_2_. FKA (99%) were isolated from kava extracts by LKT Laboratories, Inc. (St. Paul, MN, USA). Antibodies for HER2, phosphorylated AKT, AKT, Bim, survivin, XIAP, PARP, Cleaved PARP, Cdc25C, Cdc2, Wee1, Myt1, phosphorylated Cdc25C at Ser216, and phosphorylated Cdc2 at Tyr15 were from Cell Signaling Technology, Inc. (Danvers, MA, USA). Cyclin B, BAX, Bcl2, Bcl-_X/L_, and β-actin were from Santa Cruz Biotechnology, Inc. The antibody for MPM-2 was from Upstate Biotechnology (Lake Placid, NY, USA). Histone H1 was from Boehringer Mannheim, Corp. (Indianapolis, IN, USA). Protein A/G-plus agarose and protein A-plus agarose beads were from Santa Cruz Biotechnology, Inc. (Dallas, TX, USA), [γ-32P]ATP (specific activity, 3000 Ci/mmol) and enhanced chemiluminescence detection system were from Amersham Corporation (Arlington Heights, IL, USA). 3-(4,5-dimethylthiazol-2-yl)-2,5-diphenyl-tetrazolium bromide (MTT) and propidium iodide was obtained from Sigma (St. Louis, MO, USA).

### 4.2. MTT Assay

Cells were seeded onto 24 well plates at a density of 2 × 10^4^ cells for 24 h and then treated with FKA at indicated concentrations. After 72 h incubation, 200 µL of MTT solution was added to each well and incubated at 37 °C for 2 h. The MTT solution was then aspirated and 200 µL of dissolving buffer was added to each well. Cell viability was determined by measuring absorbance at 570 nm in a microplate reader (Bio-Rad, Hercules, CA, USA). Dose response curves were generated as a percentage of vehicle control treated cells using Excel software, and IC_50_ values were estimated graphically from the plot.

### 4.3. Soft Agar Colony Formation

A total number of 5000 MCF7 or MCF7/HER2 cells were seeded on the top layer containing 0.35% solidified agar in complete medium in 6-well plates and the bottom layer consisted of 0.8% agar in complete medium. Vehicle control (0.05% DMSO) or indicated concentrations of FKA in complete medium were added and replaced every three days. After three weeks of cell seeding, the number of colonies was counted under an inverted phase-contrast microscope at 100× magnification and a group of >10 cells was indicated as a colony.

### 4.4. Flow Cytometric Analysis of Cell Cycle Distribution

MCF7, MCF7/HER2, and SKBR3 cells at 70% to 80% confluency were treated with 0.1% DMSO or 16 μmol/L of FKA for 24 h. After treatment, cells were fixed in ice-cold 70% ethanol overnight. After fixation, cells were washed thrice with cold PBS and then stained in 500 μL of propidium iodide solution. Samples were analyzed on a BD FACScan flow cytometer (San Jose, CA, USA) and the percentage of cells in the S, G0-G1, and G2-M phases of the cell cycle was determined.

### 4.5. Western Blotting Analysis

Volumes of clarified protein lysates containing equal amounts of protein (50 µg) were separated on 10%–12% sodium deodecyl sulfate-polyacrylamide gel electrophoresis (SDS-PAGE) and electrophoretically transferred to Hybond-ECL membranes (GE Healthcare, Piscataway, NJ, USA). The blots were then probed with primary antibody, followed by secondary antibodies as described previously [15]. Immunoreactive bands were visualized using an enhanced chemiluminescence detection system (Thermo Scientific, Rockford, IL, USA).

### 4.6. In Vitro Kinase Assay

Cdc2-associated H1 histone kinase activity was determined as described by Zi et al. [16]. Briefly, using anti-Cdc2 antibody and protein A-agarose beads, Cdc2 was immunoprecipitated from 200 μg of protein lysate per sample, as detailed above. Beads were washed three times with lysis buffer and then once with kinase assay buffer (50 mM Tris-HCl, pH 7.4, 10 mM MgCl2, and 1 mM DTT). Phosphorylation of histone H1 was measured by incubating the beads with 40 μL of “hot” kinase solution [0.25 μL (2.5 μg) of histone H1, 0.5 μL of [γ-^32^P] ATP, 0.5 μL of 0.1 mM ATP, and 38.75 μL of kinase buffer] for 30 min at 37 °C. The reaction was stopped by boiling the samples in SDS sample buffer for 5 min. The samples were analyzed by 12% SDS-PAGE, and the gel was dried and subjected to autoradiography. 

### 4.7. 4',6-Diamidino-2-phenylindole (DAPI) Nuclear Staining

SKBR3 cells (4 × 10^4^ cells/well) were cultured on chamber slides for 24 h. The cells were then treated with different concentrations of FKA for 24 h. After treatments, the cells were rinsed in 1× PBS three times, and fixed in 4% paraformaldehyde. The fixed cells were mounted in Vector shield medium containing DAPI (Vector Laboratories, Inc., Burlingame, CA, USA) in a darkroom and visualized with a Nikon Eclipse TE2000-S (200× magnification) microscope (Tokyo, Japan) under ultraviolet light. Apoptotic cells were identified by the nuclear condensation and fragmentation.

## 5. Statistical Analysis

Comparisons of cell density, number of colonies and relative levels of protein expression between the different treatments were conducted using Student’s *t*-test. All statistical tests were two-sided. *p* < 0.05 was considered statistically significant.

## Figures and Tables

**Table 1:** The IC_50s_ of FKA and statuses of estrogen receptor, p53, and HER2 in breast cancer cell lines.

	MCF10A	MDA-MB468	MCF7	MCF7/HER2	SKBR3
**Estrogen receptor**	absent	mutant	wild-type	wild-type	mutant
**P53 **	wild-type	mutant	wild-type	wild-type	mutant
**HER2 expression**	+	+	+	++++	++++
**IC_50s_**	>100 μM	38.4 μM	45 μM	13.6 μM	10 μM

Note: “+”, weak positive; “++++”, strong positive.
